# Vonoprazan attenuates proteinuria in diabetic kidney disease through potential direct renal mechanism

**DOI:** 10.1038/s41598-025-25376-8

**Published:** 2025-11-21

**Authors:** Rabab Mahmoud Ahmed, Amin Roshdy Soliman, AbdelAal Mohammed

**Affiliations:** Internal Medicine and Nephrology Department, Kasr Alainy Faculty of Medicine, Cairo University, Cairo, Egypt

**Keywords:** Vonoprazan, Diabetes, Albuminuria, H-pylori, Endocrinology, Medical research, Nephrology

## Abstract

Our study investigates the effect of vonoprazan on albuminuria in diabetic kidney disease (DKD) patients compared to lansoprazole through a randomized controlled trial. Participants were randomized to receive either lansoprazole 30 mg or vonoprazan 20 mg. Specifically, 100 patients were included for H. pylori eradication, and 40 patients for other indications. The albumin/creatinine ratio (ACR) and estimated glomerular filtration rate (eGFR) were measured at baseline and three months later. Both vonoprazan and lansoprazole significantly reduced ACR from baseline levels after 3 months (*p* < 0.05). However, the reduction in albuminuria was significantly greater in the vonoprazan group compared to the lansoprazole group, regardless of H. pylori diagnosis or eradication success (*p* < 0.05). Treatment type was a significant predictor, with a standardized beta of 0.555. Patients receiving vonoprazan experienced a 19.2-unit increase in the percentage change of ACR compared to those receiving lansoprazole (β = 19.219, 95% CI [13.62-24.817]). Additionally, vonoprazan-treated patients showed significant improvement in eGFR after 3 months compared to baseline levels (*p* < 0.05), with this improvement being superior to that observed with lansoprazole (*p* < 0.05). Vonoprazan may have a direct renal effect, warranting further research to elucidate its mechanism in reducing albuminuria.

## Introduction

 Proton pump inhibitors (PPIs) have been linked to acute kidney injury (AKI), chronic kidney disease (CKD), and progression of existing kidney disease. Despite this, (PPIs) are widely used as acid inhibitory agents to treat gastroesophageal-related disorders in patients with diabetic kidney disease (DKD), who often receive multiple medications which can cause gastric irritation or ulcers^1^.

Diabetic kidney disease (DKD) is one of the most prevalent complications of diabetes, affecting approximately 40% of cases, and is the leading contributor to end-stage renal disease (ESRD)^2^. Although therapeutic strategies have advanced, the reduction of proteinuria remains a critical yet evolving goal in nephrology, requiring further investigation to optimize outcomes. Zhang et al. (2024) highlight the need for multidrug approaches reinforcing the call for continued research into effective proteinuria-lowering therapies. The promising effects of combination therapy with two or more agents in reducing albuminuria and preserving renal function warrant further exploration^3^.

Lansoprazole stands out from other (PPIs) due to its unique pharmacological properties, such as potential anti-inflammatory effects, reduced oxidative stress, anticancer potentials, and anti-diabetic benefits^4^. There are conflicting results regarding PPI use and the subsequent risk of developing or progressing albuminuria or eGFR decline in patients with diabetes^5–9^. However, various studies have demonstrated that eradicating H. pylori using triple therapy consisting of lansoprazole, amoxicillin, and clarithromycin significantly reduces proteinuria in diabetic, non-diabetic, and primary glomerulonephritis patients^10–13^.

In May 2022, the FDA approved the use of the potassium-competitive acid blocker vonoprazan as an alternative antisecretory agent for the treatment of H. pylori infection and other gastrointestinal disorders^14^. No data exist in the literature regarding its effect on proteinuria.

The aim of this study is to determine whether vonoprazan has a differential effect on albuminuria in patients with DKD compared to lansoprazole.

## Materials and methods

### Study design and patient’s enrolment

This is a a prospective randomized controlled single blinded parallel trial conducted at the outpatient clinics of Cairo University Hospital on patients with DKD recruited between January 2024 and April 2024. **The study was registered in Pan African Clinical Trails Registry at (25/11/2024) with registration ID number: PACTR202411502424899 and CONSORT reporting guidelines was also used to complete the CONSORT Checklist**^15^.

#### Ethical committee approval

This study was approved by the Research Ethics Committee of the Faculty of Medicine at Cairo University in December 2023 (approval number: **N-394–2023**). Informed written consent was obtained from all participants.

### Randomization method

randomly assigning patients, ensuring a 1:1 allocation ratio for both study groups. Allocation Concealment was maintained by using sealed, opaque envelopes to prevent selection bias. Stratification and Blocking: The protocol does not specify any stratification or blocking procedures for the randomization process.

### Blinding procedures

For this single-blinded study, participants, data collectors, and those assessing the outcomes were blinded to the treatment assignments, but the health care providers are not blinded. Blinding was maintained throughout the study by ensuring consistent handling procedures for both treatment groups. Any information that could reveal the group allocation was concealed from participants, data collectors, and outcome assessors. Unblinding Procedures: will only conducted under specific conditions where it was necessary, such as in the case of serious adverse events requiring knowledge of the treatment allocation.

### Study protocol

#### Inclusion criteria

**Fig. 1:**
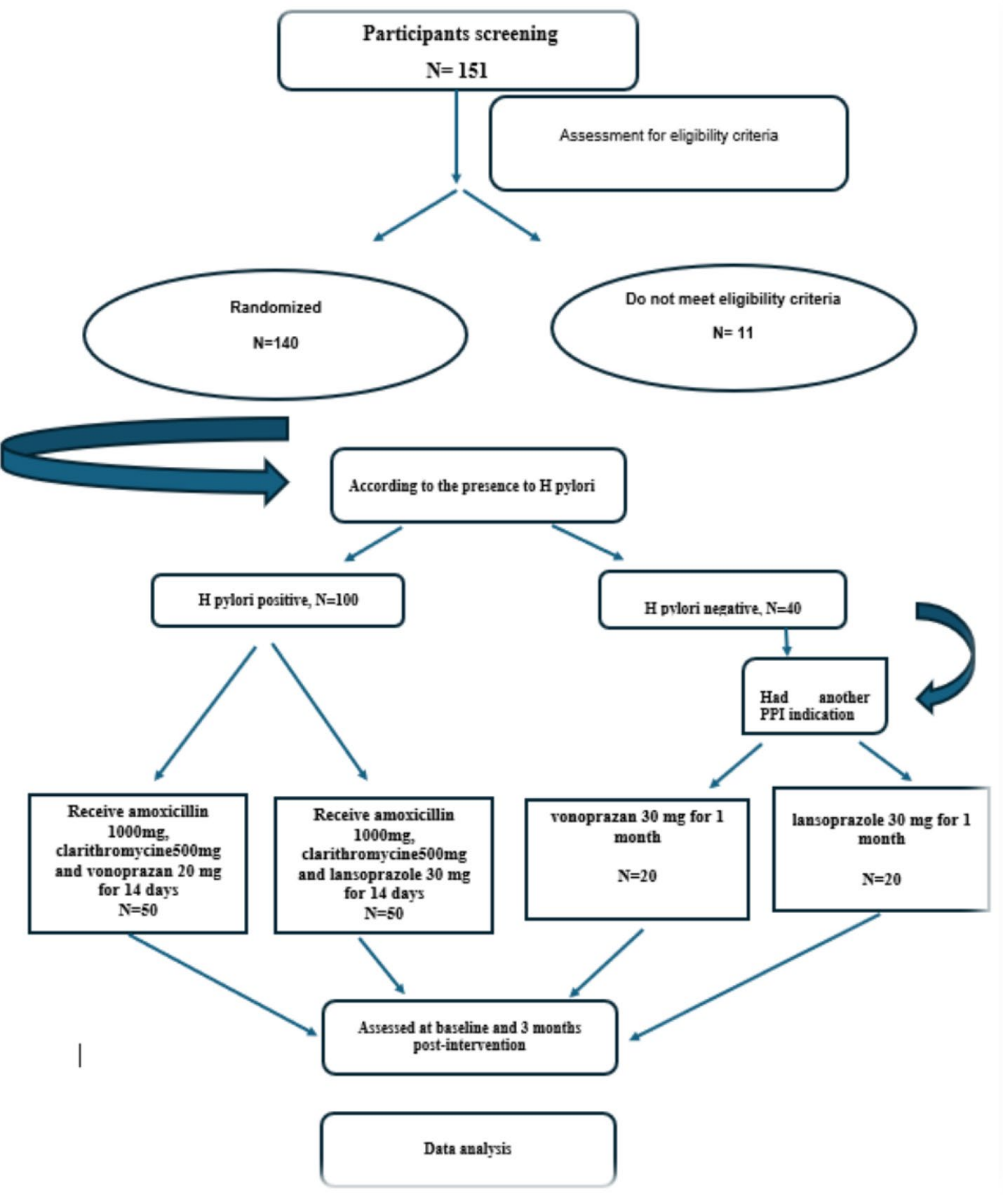
Study flow chart.

Patients with DKD who had an albumin/creatinine ratio exceeds 500 mg/day for at least six months before inclusion and a glomerular filtration rate (GFR) greater than 30 ml/min were enrolled. The albuminuria threshold (> 500 mg/day) was sustained throughout the six-month period and reconfirmed at the time of enrolment. In the initial phase, 100 patients with (DKD) who Met the ACR and eGFR criteria and had a confirmed Helicobacter pylori infection were included. In the subsequent phase, an additional 40 DKD patients with the same ACR and eGFR criteria were enrolled based on their prescription of proton pump inhibitors (PPIs) for indications unrelated to H. pylori infection. All patients were receiving stable treatment with either the maximum tolerated dose of ACE inhibitors or ARBs. 151 DKD patients were assessed for eligibility criteria, of them 140 patients were randomized. The flowchart is presented in Fig. 1

#### Exclusion criteria

Patients with malignancies, liver disease, cardiac failure, acute coronary syndrome, pregnancy, lactation, active infection within the last two months, a history of acid-suppressive drug use within the last three months, a history of upper gastrointestinal system disease or surgery, previous eradication therapy for H. pylori, use of SGLT2 inhibitors and other drugs that can affect proteinuria, a GFR less than 30 ml/min, uncontrolled hypertension requiring new or changing doses of antihypertensive drugs within the last three months, and those unable to provide informed consent.

The inclusion of patients in this study was conducted in two stages. Initially, 100 patients diagnosed with DKD and positive H. pylori were included. These patients were randomly assigned (1:1) using sealed envelopes, with 50 patients in each group. One group received amoxicillin 1000 mg, clarithromycin 500 mg, and lansoprazole 30 mg twice daily, while the other group received vonoprazan 20 mg, amoxicillin 1000 mg, and clarithromycin 500 mg twice daily. All patients were treated for 14 days as eradication therapy.

The patients in each group were further divided into two subgroups based on their response to the H. pylori eradication treatment into (Stool H. pylori antigen negative or Stool H. pylori antigen positive).

For these 100 patients, the following were recorded: age, sex, body mass index (BMI), systolic and diastolic blood pressure, serum creatinine, estimated glomerular filtration rate (eGFR), serum albumin, total protein, urinary albumin/creatinine ratio (mg/g), HbA1c, fasting blood glucose, and 2-hour postprandial blood glucose. These measurements were taken at baseline and again after three months after treatment.

Next, we included an additional 40 patients with DKD but had another indication for PPI. These patients were randomly assigned to receive either lansoprazole 30 mg or vonoprazan 20 mg for one month, with 20 patients in each group. The same randomization method described earlier was used. For these 40 patients, the albumin/creatinine ratio (ACR) and estimated glomerular filtration rate (eGFR) were measured at baseline and after three months.

The expected duration of participation for each subject was 4 months, including a screening (baseline) period, a treatment period and a follow-up period.

This approach allowed us to evaluate the effect of these drugs on albuminuria, independent of the presence of H. pylori or eradication therapy.

In Egypt, vonoprazan is available as a standalone drug, unlike in the USA where it is only available in combination with other H. pylori eradication therapies.

#### Safety considerations

Any adverse event (AE) observed by the study personnel or reported by the participant will be recorded promptly. Participant’s unique identifier, Date and time of the event, Description of the event, Severity (mild, moderate, severe), Duration (start and end dates), Relationship to study medication (related, possibly related, unrelated), Actions taken (medication adjustment, additional treatment, study withdrawal), Outcome (resolved, ongoing, unresolved) will be documented.

The trial is being conducted in compliance with the Declaration of Helsinki. All participants gave written informed consent prior to any trial-related activities.

#### Outcome measured

##### *Primary outcome*

The change in ACR from baseline and after 3 months of treatment.

##### *Secondary outcome*

The change in eGFR from baseline and after 3 months of treatment.

The estimated glomerular filtration rate (eGFR) was calculated using the 2021 CKD-EPI (Chronic Kidney Disease Epidemiology Collaboration) formula: [eGFR = 142×min⁡(Scr/κ,1)^α^×max⁡(Scr/κ,1) ^−1.200^ × 0.9938 ^Age^× Sex Factor]^16^.

Variables: Scr = serum creatinine (mg/dL), κ (kappa) = 0.7 for females, 0.9 for males, α (alpha) = − 0.241 for females, − 0.302 for males, Sex Factor = 1.012 for females, 1.000 for male, Age = age in years, min(Scr/κ, 1) = the smaller of Scr/κ or 1, max(Scr/κ, 1) = the larger of Scr/κ or 1.

H. pylori was diagnosed by stool antigen test at baseline and two weeks after treatment completion. Normoalbuminuria is defined as Urinary albumin-to-creatinine ratio (UACR) less than 30 mg/gCr, Microalbuminuria: Urinary albumin-to-creatinine ratio (UACR) between 30 and 300 mg/gCr and Macroalbuminuria: Urinary albumin-to-creatinine ratio (UACR) greater than 300 mg/gCr^17^.

##### Sample size estimation

This study aimed to evaluate the effects of the newly FDA-approved drug vonoprazan on proteinuria and to compare its effect with lansoprazole. As this intervention is new and has never been used before, it will be a pilot study. The suggested total sample size is 100 (50 per group). According to Connelly (2008), extant literature suggests that a pilot study sample should be 10% of the sample projected for the larger parent study. However, Hertzog (2008) cautions that this is not a simple or straightforward issue to resolve because these types of studies are influenced by many factors. Treece and Treece (1982) also suggested 10% of the project sample size^18^.

##### Statistical analysis

Analysis of data was done by IBM computer using SPSS (statistical program for social science version 23) as follows: Description of quantitative variables as Mean, SD, Median and IQR according to shapiro test of normality. Description of qualitative variables as number and percentage. Chi-square test was used to compare qualitative variables between groups. Fisher exact test was used when one expected cell or more are less than 5.Independent t test was used instead of to compare quantitative variables between two groups in parametric data Mann Whitney test was used instead of unpaired t-test in non-parametric data (SD > 30%mean). Wilcoxon signed rank test used to compare quantitative data before and after treatment Backward Linear regression used to find predictors for readmission. P value > 0.05 was considered insignificant, *P* < 0.05 was considered significant.

## Results

A total of 140 patients were included in this study.

### The 100 patients with DKD & positive H pylori

The mean age of the participants was 53.24 ± 10.87 years (female: 46%; male: 54%). Demographic and laboratory findings were comparable at baseline, as shown in Table 1. No reported adverse effects in both groups. After 3 months of follow-up, patients who received either vonoprazan or lansoprazole as part of H. pylori eradication therapy exhibited significant reductions in albuminuria, fasting blood glucose (FBG), 2-hour postprandial glucose (2 h PPG), and HbA1c levels (*p* ≤ 0.05). However, vonoprazan demonstrated superior improvement in serum creatinine and corresponding eGFR compared to lansoprazole, with statistical significance (*p* < 0.001). 72% of patients treated with Vonoprazan tested negative for H. pylori, compared to 56% of those treated with Lansoprazole. While Vonoprazan appears to have a higher percentage of negative results compared to Lansoprazole, the difference is not statistically significant, *p* = 0.096.

**Table 1:** Comparison of baseline demographic and laboratory data with 3-Month Follow-Up data for each studied group in H pylori positive patients.

	Vonoprazan (50 patients)					Lansoprazole (50 patients)				p value
**Age** (Independent t test)										
**Mean ± SD**	53.46 ± 11.55					53.02 ± 10.27				0.841
**Range**	30–79					34–77				
**Sex**,** N (%) (**Chi square**)**										
**Male**	27(54)					27(54)				1
**Female**	23(46)					23(46)				

	**Baseline**		**3months follow up**			**Baseline**		**3months follow up**		
	**Median(IQR)**	**Range**	**Median(IQR)**	**Range**	**p value**	**Median(IQR)**	**Range**	**Median(IQR)**	**Range**	**p value**
**BMI**	27(26–29)	22–36	27(25–30)	22–36	0.275	27(26–28)	24–33	27(26–28)	24–34	0.276
**SBP**	130(120–135)	110–155	130(120–135)	100–150	0.478	130(125–135)	120–170	130(125–135)	120–150	0.394
**DBP**	80(70–85)	60–100	80(70–90)	60–100	0.541	75(70–80)	70–110	80(75–90)	60–100	0.312
**creatinine**	1.7(1.5–1.8)	1.3–2.3	1.6(1.4–1.8)	1.2–2.3	**< 0.001**	1.7(1.6–1.8)	1.5–1.9	1.7(1.6–1.8)	1.4–1.9	0.288
**Total protein**	7(6.8–7.2)	5.9–7.9	7(6.9–7.1)	6–7.7.7	0.752	7(6.9–7.3)	6.2–8.2	7(7–7.2.2)	6.4–8.4	0.74
**Albumin**	4.1(3.9–4.3)	3.5–4.9	4(4–4.2.2)	3.4–4.9	0.934	4(3.8–4.1)	3.3–4.7	4(3.9–4.1)	3.3–4.8	0.099
**ACR**	1188(800–1369)	600–2735	819.5(512–987)	237–1951	**< 0.001**	1120(877–1333)	600–3345	984(785–1211)	314–3211	**< 0.001**
**FBS**	129(122–143)	100–198	122.5(113–130)	100–143	**< 0.001**	140(125–166)	120–199	133.5(125–154)	113–177	**0.006**
**2hpp Blood sugar**	165.5(145–189)	56–276	145.5(130–160)	34–201	**< 0.001**	168.5(155–211)	133–297	158.5(144–176)	124–220	**< 0.001**
**HbA1c**	7.2(7.1–7.5)	6.5–8.3	7.1(7–7.3.3)	6.5–7.9	**< 0.001**	7.1(7–7.5.5)	6.5–8.3	7.1(7–7.3.3)	6.4–8.4	**0.032**
**GFR**	42.5(38–47)	31–63	44.5(39–51)	30–82	**< 0.001**	43.5(35–50)	30–62	43.5(37–50)	31–66	0.482
**Response to treatment: Stool H. pylori antigen after eradication therapy**										
**Negative N (%)**		36(72)				28(56)				0.096
**Positive N (%)**		14(28)				22(44)				

Wilcoxon test is the test of significance p value significant if ≤ 0.05.

BMI = body mass index; SBP = systolic blood pressure; DBP = diastolic blood pressure; TP = serum total protein; Alb = serum albumin; ACR = Albumin creatinine ratio; FBG = fasting blood sugar; 2hppBG = 2 h post prandial blood sugar.

Overall, the percentage decrease in albuminuria, regardless of treatment success, was significantly higher in the vonoprazan group compared to the lansoprazole group (30.85 vs. 8.88, *p* < 0.001). Among patients successfully treated for H. pylori (negative), the decrease in albuminuria was also significantly higher in the vonoprazan group (30.39 vs. 7.26, *p* < 0.001). Even among patients who remained H. pylori positive, vonoprazan still outperformed lansoprazole significantly (*p* = 0.001). (Table 2).

**Table 2:** Percent change decrease in albuminuria in Vonoprazan containing H pylori therapy vs Lansoprazole containing H pylori therapy.

Group II (vonoprazan) N = 50			Group I (lansoprazole) *N* = 50		p valve
	**Median (IQR)**	**Range**	**Median (IQR)**	**Range**	
**Total ACR percent change decrease in all patients**	30.85(21.98–37.5)	−7.44-64.37	8.88(3.11–20.12	−21.58-47.67	**< 0.001**
**ACR percent change decrease in H pylori Ag negative after eradication**	30.39(22.69–37.24)	−7.44-53.1	7.26(3.28–18.31)	−13.54-47.67	**< 0.001**
**ACR percent change decrease in H pylori Ag positive after eradication**	33.57(21.59–48.85)	0.66–64.37	9.61(3.11–21.91)	−21.58-37.33	**0.001**

Within each group and the entire H pylori study population, there was no statistically significant difference in the decrease in albuminuria % change decrease after eradication treatment between those who remained H. pylori positive and those who became negative, (Table 3).

**Table 3:** Effect of eradication status on ACR present change decrease in the whole H pylori study population and within each group.

Stool H.pylori Ag					P value
	Negative		Positive		
	Median(IQR)	Range	Median(IQR)	Range	
ACR percent change decrease in the whole H pylori study population regardless drug type	22.69(6.18- 34.38)	−13.54- 53.1	19.73(6.41- 30.74)	−21.58- 64.37	0.672
ACR percent change decrease within Vonoprazan group	30.39(22.69–37.24)	−7.44-53.1	33.57(21.59–48.85)	0.66–64.37	0.503
ACR percent change decrease within Lansoprazole group	7.26(3.28–18.31)	−13.54-47.67	9.61(3.11–21.91)	−21.58-37.33	0.635

We conducted Backward multiple linear regression model c to find predictors for ACR percentage of change. The age, BMI and treatment Type were found to be the significant predictors among variables entered in the model. Treatment type was found to be the highly significant predictor where standardized beta was 0.555. patients receiving Vonoprazan were more liable to increase by 19.2 units in Percentage of change of ACR in comparison to Lansoprazole (β = 19.219 95th CI (13.62–24.817.62.817)) While increase in Age and BMI was associated with decrease in the percent of change of ACR (β −0.327 95th CI (−0.592-0.062) and − 1.896 95th CI(−3.063-0.729) respectively), Table (4).

**Table 4:** Predictors of decrease in albuminuria.

Unstandardized Beta Coefficients		Standardized beta Coefficients	*p* value	95% CI for B		Collinearity Statistics
**(Constant)**	80.533		0	42.994	118.072	
**Age**	−0.327	−0.205	0.016	−0.592	−0.062	0.946
**BMI**	−1.896	−0.27	0.002	−3.063	−0.729	0.94
**Treatment type(Vono-prazan)**	19.219	0.555	< 0.001	13.62	24.817	0.992

Backward multiple linear regression model c. Dependent Variable: albuminuria percent change decrease. Independents: treatment type (vonoprazan versus lansoprazole), Age, H.pylori antigen, sex and baseline BMI. CI confidence interval R square 0.368.

### The 40 patients with DKD & other PPI indications

After 3 months of follow-up, no reported adverse effects in both groups. There was no statistically significant difference in the reduction of albuminuria between Vonoprazan and Lansoprazole (*p* = 0.076). Both drugs significantly reduced the Albumin-to-Creatinine Ratio (ACR) from baseline levels compared to 3 months post-treatment (*p* < 0.05). However, the percentage reduction in ACR was significantly greater in the Vonoprazan group (*p* < 0.001, Table 5). Additionally, patients treated with Vonoprazan showed a significant improvement in their estimated Glomerular Filtration Rate (eGFR) after 3 months compared to baseline levels (*p* = 0.017), and this improvement was superior to that observed with Lansoprazole, with statistical significance (*p* = 0.001), Table 5.

**Table 5:** Effect of Vonoprazan vs Lansoprazole on ACR &eGFR in patients with DN & dyspepsia.

	Vonoprazan N = 20	Lansoprazole N = 20	*P value
**ACR at baseline**			
Median(IQR)	3164.5(1472–4046)	1935(1171.5–2723)	0.063
Range	900–5682	660–5674	
**ACR after 3 m**			
Median(IQR)	1009(820–1900)	1700(1107.5–2585)	0.076
Range	567–4100	655–4700	
**#P value**	**< 0.001**	**0.003**	
**ACR percent change decrease**			
Median(IQR)	49.25(27.7–66.9)	2.65(0.5–12.85.5.85)	**< 0.001**
Range	−8.2-80.9	−4.2-24.3	

	**Vonoprazan**	**Lansoprazole**	*P value
GFR at baseline			
Median(IQR)	47(43.5–53)	40(36.5–46)	0.057
Range	33–67	33–59	
GFR 3 m			
Median(IQR)	49.5(44–55.5.5)	40(35.5–49)	**0.001**
Range	38–60	34–55	
**#P value**	**0.017**	0.55	

*Mann Whitney U test # Wilcoxon test p value significant ≤ 0.05.

## Discussion

There is no prior evidence or specific studies linking vonoprazan to improvements or deteriorations in proteinuria associated with diabetic kidney disease. In our study, patients with diabetic kidney disease and positive H. pylori showed a significant reduction in the Albumin-to-Creatinine Ratio (ACR) from baseline levels after 3 months of treatment with either vonoprazan or lansoprazole. This effect is logical and supported by numerous studies indicating that the eradication of H. pylori significantly reduces proteinuria in patients with diabetic kidney disease^9,10^.

The type of drug used for eradication in our study resulted in different outcomes. The reduction in albuminuria was significantly higher in the vonoprazan group compared to the lansoprazole group. This can be attributed to vonoprazan’s enhanced efficacy on H+,K + ATPase, which is approximately 350 times more potent than that of lansoprazole^19^.

This increased potency not only appears in the magnitude of proteinuria reduction but also in the higher percentage of treated patients achieving eradication compared to lansoprazole in our study.

New evidence from our research indicates that vonoprazan decreased albuminuria regardless of H. pylori eradication status. This effect was also observed in patients who received vonoprazan for indications other than H. pylori infection, suggesting a unique direct renal effect of this drug class on albuminuria in patients with diabetic kidney disease.

While H-K ATPase is not directly associated with albumin excretion, we propose that vonoprazan may have an effect similar to SGLT2 inhibitors, considering that renal ion transporters are integral to the complex pathophysiological mechanisms of diabetic kidney disease (DKD) and the recent clinical benefits of sodium–glucose cotransporter 2 (SGLT2) inhibitors in DKD highlight the crucial role of renal ion homeostasis in disease progression^20,21^.

Potassium channels within the renal tubules are vital in the mechanisms that lead to proteinuria occurrence or progression in DKD. Alterations in K + channel activity can impact hemodynamic resistance, kidney blood flow, and glomerular filtration pressure, thereby affecting protein excretion in the urine. These channels help mitigate angiotensin-related podocyte injury and intrarenal pressure, and inhibit TGF-β1 signalling, thus reducing proteinuria and kidney injury^18^. Recent evidence suggests that impaired potassium balance in nephrotic syndrome is related to proteinuria^22–24^.

Ensuring optimal kidney function through proper acid-base and electrolyte balance can potentially alleviate stress on the glomeruli, indirectly impacting albuminuria levels.

Vonoprazan may have similar effects to lansoprazole (LPZ), such as potential anti-inflammatory properties, reduced oxidative stress, and anti-diabetic benefits, which could lead to a reduction in albuminuria^4,19^. This might explain the significant improvement in fasting blood glucose, 2-hour postprandial glucose, and HbA1c levels observed in our patients receiving vonoprazan and lansoprazole.

Despite current clinical treatments for diabetic kidney disease (DKD) that aim to alleviate kidney damage by addressing direct and indirect metabolic and hemodynamic factors, such as using SGLT2 inhibitors to improve proteinuria and eGFR in patients with CKD^25^, our study cannot attribute albuminuria reduction to these drugs. This is because we excluded patients using SGLT2 inhibitors and other drugs that can affect proteinuria from our enrollment criteria.

The mechanisms of action for vonoprazan and other PPIs are entirely different, evident by the improvements in serum creatinine and corresponding eGFR in vonoprazan groups, compared to lansoprazole, despite the known risk of PPI nephrotoxicity^19^.

However, the renal effects of vonoprazan cannot be fully explained by these suggested mechanisms. Further research is necessary to elucidate the precise role of H-K ATPase in diabetic kidney disease and its potential impact on albuminuria. Understanding these mechanisms could pave the way for new therapeutic interventions. Future researches explore the molecular, mechanistic, and pharmacological basis of vonoprazan’s effect on proteinuria in diabetic kidney disease are recommended. While the current findings are clinically intriguing, a deeper understanding of the drug’s renal actions at the cellular and biochemical levels would provide valuable insight and strengthen the translational relevance of this work.

### Strengths and limitations

This study is the first to explore the effect of the newly approved drug vonoprazan on albuminuria in diabetic kidney disease. However, the relatively small sample size and unmeasured confounders remain limitations especially in H Pylori negative patients.

### Conclusion

Our work suggests that vonoprazan has pleiotropic beneficial renal effects beyond its gastrointestinal benefits. Further research is needed to elucidate the exact mechanism by which vonoprazan decreases albuminuria in DKD.

## Data Availability

The datasets used and/or analysed during the current study available from the corresponding author on reasonable request.
